# Dominance style only partially predicts differences in neophobia and social tolerance over food in four macaque species

**DOI:** 10.1038/s41598-020-79246-6

**Published:** 2020-12-16

**Authors:** Federica Amici, Anja Widdig, Andrew J. J. MacIntosh, Victor Beltrán Francés, Alba Castellano-Navarro, Alvaro Lopez Caicoya, Karimullah Karimullah, Risma Illa Maulany, Putu Oka Ngakan, Andi Siady Hamzah, Bonaventura Majolo

**Affiliations:** 1 Behavioral Ecology Research Group, Institute of Biology, Faculty of Life Science, University of Leipzig, Leipzig, Germany; 2 Research Group Primate Behavioural Ecology, Department of Human Behavior, Ecology and Culture, Max-Planck Institute for Evolutionary Anthropology, Leipzig, Germany; 3 Primate Research Institute, Kyoto University, Aichi, Japan; 4 Fundació Universitat de Girona, Innovació I Formació, Girona, Spain; 5 Ethology and Animal Welfare Section, Universidad Cardenal Herrera-CEU, CEU Universities, Valencia, Spain; 6 Department of Clinical Psychology and Psychobiology, Faculty of Psychology, University of Barcelona, Barcelona, Spain; 7 School of Biological Sciences, Universiti Sains Malaysia, Pulau, Pinang Malaysia; 8 Forestry Department, Hasanuddin University, Makassar, Sulawesi Indonesia; 9 School of Psychology, University of Lincoln, Lincoln, UK

**Keywords:** Psychology, Zoology

## Abstract

Primates live in complex social systems with social structures ranging from more to less despotic. In less despotic species, dominance might impose fewer constraints on social choices, tolerance is greater than in despotic species and subordinates may have little need to include novel food items in the diet (i.e. neophilia), as contest food competition is lower and resources more equally distributed across group members. Here, we used macaques as a model to assess whether different dominance styles predict differences in neophilia and social tolerance over food. We provided familiar and novel food to 4 groups of wild macaques (N = 131) with different dominance styles (*Macaca fuscata*,* M. fascicularis*,* M. sylvanus*,* M. maura*). Our study revealed inter- and intra-specific differences in individuals’ access to food, which only partially reflected the dominance styles of the study subjects. Contrary to our prediction, social tolerance over food was higher in more despotic species than in less despotic species. Individuals with a higher dominance rank and being better socially integrated (i.e. higher Eigenvector centrality) were more likely to retrieve food in all species, regardless of their dominance style. Partially in line with our predictions, less integrated individuals more likely overcame neophobia (as compared to more integrated ones), but only in species with more tolerance over food. Our study suggests that individual characteristics (e.g. social integration or personality) other than dominance rank may have a stronger effect on an individual’s access to resources.

## Introduction

One of the consequences of group-living is that individuals compete with other group members to attain a dominant position and have preferential access to fitness-related resources. The type and intensity of such within-group competition, and the role of dominance rank in shaping social interactions between group members differ across populations and species. Group-living species display different dominance styles, which vary along a gradient from more to less despotic depending, among other factors, on the symmetry of their agonistic interactions and their conflict management patterns^1–4^. In less despotic species, for instance, aggressive conflicts are expected to have a greater frequency of counter-aggression and more undecided outcomes, higher reconciliation and shallower dominance hierarchies than more despotic species^4,5^. Moreover, dominance might impose fewer constraints on social choices in less despotic species, and individuals might have more opportunities to interact with many different social partners^6–11^.

Although several studies have investigated the relationship between dominance style, aggressive and affiliative behaviour, much less is known about the potential link between dominance style and other important aspects of animal behaviour. Access to resources, for instance, is crucial for all individuals and species, as food provides essential energy to live and reproduce^12,13^. Usually, access to food is predicted by individual dominance rank, as higher-ranking individuals usually have priority of access to resources^14–20^. Similarly, access to food may be predicted by an individual integration in the social network, as highly gregarious individuals and/or those individuals connected to highly gregarious partners^21^ might rely on a larger network of social partners and thus experience higher tolerance near food^22^. In less despotic species, however, the importance of rank and social integration in a food acquisition context may be minor compared to despotic species, because subordinates or individuals with weaker social integration may nonetheless receive a higher share of resources, as they may try to access food without a high risk of aggression from more dominant group members^23–25^. Although tolerance over food might be higher in less despotic species (see^23–26^), there are few studies directly comparing access to food sources across species with different dominance styles^27,28^.

Another factor likely to differ depending on the species dominance style is the degree of neophobia, that is, the degree to which individuals avoid novelty^29^. Clearly, no stimulus can be completely novel, as it necessarily shares features (e.g. colour, shape, consistency, odour) with other familiar stimuli that can be generalized to it. In this respect, neophobia should be considered a relative term measuring variation in the response to stimuli of varying familiarity, which we will use here for consistency with previous work. Low levels of neophobia toward novel food, for example, may facilitate exploration and innovation, but they may also expose individuals to novel risks when exploring unfamiliar food^30–34^. Depending on the degree of food competition experienced and on the ease with which they can access food, therefore, individuals may show different levels of neophobia, overcoming their neophobic tendencies when resources are scarce^35–42^. By usually having better access to resources^43^, for instance, higher-ranking individuals may gain lower potential payoffs from novelty, and thus be more neophobic^32,44–48^. Similarly, individuals with better social integration in the group might have higher fitness (see e.g.^49–55^), as social integration might favour tolerance and/or support against other competing individuals. Therefore, socially integrated individuals might also obtain lower potential payoffs from novelty (see^48^). If rank and social integration are linked to differences in neophobia, dominance style might play a crucial role to modulate these effects. In more despotic species, food distributions may be less equitable, and less dominant or less integrated individuals may have to more strongly rely on novel, riskier food to survive. To our knowledge, however, no study has directly tested whether different dominance styles affect inter-individual variation in levels of neophobia.

Given these premises, it is clear that comparing species with different dominance styles is necessary to understand whether and how individual differences in neophobia and social tolerance over food are affected by species-specific differences in dominance style, and the role of these differences in modulating the effect of dominance rank and social integration across species. In this study, we used comparative data on four wild populations of macaques to test the link between dominance style, social tolerance and neophobia in a food context. Macaques are well suited for such a comparison, because they are a monophyletic group with a similar social structure, including multi-male multi-female groups, male dispersal and female philopatry, as well as matrilineal dominance hierarchies^2,4,5^. Nonetheless, macaques vary considerably in their dominance style and have been clustered into four grades, from more despotic (grade 1) to less despotic species (grade 4)^4,5^. Therefore, macaques constitute an ideal model for comparative research on sociality, by allowing to test for the effect of dominance style, while indirectly controlling for phylogeny.

We studied four wild groups of macaques, each belonging to a different grade of dominance style: Japanese macaques (*Macaca fuscata*, grade 1, i.e. highest degree of despotism; hereafter, JM), long-tailed macaques (*M. fascicularis*, grade 2; LM), Barbary macaques (*M. sylvanus*, grade 3; BM) and moor macaques (*M. maura*, grade 4, i.e. lowest degree of despotism; MM). We used standardized experimental setups by providing food to each study group in different conditions, to test inter- and intra-specific differences in access to food resources. Firstly, given that dominants in more despotic species may better monopolize resources, we predicted that the proportion of individuals having access to food in each session would be higher in less despotic than in more despotic species (Prediction 1). Secondly, we predicted that more dominant and more central individuals (i.e. with stronger connections in the social network^21,56^) would likely gain better access to food, but this effect would be stronger in more despotic than in less despotic species (Prediction 2). Finally, we predicted that, in the presence of novel food, the tendency to overcome neophobia would be higher in less dominant and less central individuals (who may otherwise have little access to food), but this effect would be stronger in more despotic than in less despotic species (Prediction 3), as access to food in the former may be more uneven across group members.

## Methods

### Ethics

All experimental protocols were approved by the ethics committees of the Kyoto University Wildlife Research Center and the City of Kushima Agency for Cultural Affairs in Japan, by the Kementarian Negara Riset dan Teknologi Republik in Indonesia (RISTEK), and by the Helping Hand Trust in Gibraltar. The study was mainly observational and all study groups were used to occasionally receiving food (see below). No further permits were required. The study was carried out in accordance with the national regulations of all the countries where the study was conducted.

### Subjects

We studied four macaque species living in wild populations, selected to represent the four dominance styles described by Thierry^4,5^. We tested one group of 55 Japanese macaques (JM) on Koshima island in Japan, one group of 26 long-tailed macaques (LM) in the Kuala Lumpur district in Malaysia, one group of 20 Barbary macaques (BM) on the cliffs of Gibraltar, and one group of 42 moor macaques (MM) on South Sulawesi in Indonesia. All groups included males and females of different age classes and dominance ranks (see Supplementary Materials and Table S1 for more details on the study animals). In all groups, monkeys exploited natural food, but they were also partially provisioned with small quantities of vegetables and fruit by human tourists (see Supplementary Materials).

### Materials and procedures

We conducted behavioural observations on all the individuals in each group, except for infants (JM: *N* = 53; LM: *N* = 26; BM: *N* = 19; MM: *N* = 33) during periods when experiments were not administered. For each species, we used the Elo method to assess the dominance hierarchy (EloRating package, version 0.46.11) and obtain the individual scaled Elo-rank (Table S1). Elo-ranks were based on all witnessed dyadic agonistic interactions (see Supplementary Material) with a clear winner-loser outcome (JM: *N* = 2116; LM: *N* = 1627; BM: *N* = 126; MM: *N* = 346), recorded via all occurrence sampling^57,58^. Dyadic agonistic interactions included aggressive interactions (i.e., bite, chase, threat, lunge), unidirectional agonistic expressions (i.e. open mouth, displacement) and unidirectional submissive behaviours (i.e. make room, bared-teeth display). The individual start values and the k factor (a weighted constant based on the probability of winning) were set by default at 1000 and 100, respectively. We then averaged the values obtained through the study periods, standardizing them to range from 0 to 1 (i.e. 0 being the lowest and 1 the highest rank). Below, we refer to these values as Elo-ranks. Elo-ranks were very stable through the study period, so we included no burn in periods. We assessed rank stability using the stab_elo function and visually inspecting the Elo-ranks (see^58^). The Elo-ranks were very stable in all study groups (JM: 0.988; LM: 0.989; BM: 0.992; MM: 0.988).

Furthermore, we assessed the spatial proximity network for each study group, based on data collected with instantaneous scans made once an hour, recording the spatially closest individual (“nearest neighbour”) of each group member. We then built an undirected weighted matrix and ran social network analyses with the following R packages: vegan (version 2.5–3^59^), asnipe (version 1.1.10^60^), and igraph (version 1.2.1^61^). We further used social network analyses to measure individuals’ Eigenvector centrality (Table S1), which is proportional to the sum of the centralities of an individual’s neighbours and assesses the importance of individuals as social hubs^21,56^. Individual Eigenvector centrality scores ranged from 0 to 1. We recorded 199 h of scan data in JM, 139 h in LM, 61 h in BM, and 229 h in MM. The lower number of observations hours for BM is due to the fact that the study group lived on a cliff, and could not be followed as much as the other groups.

**Figure 1:**
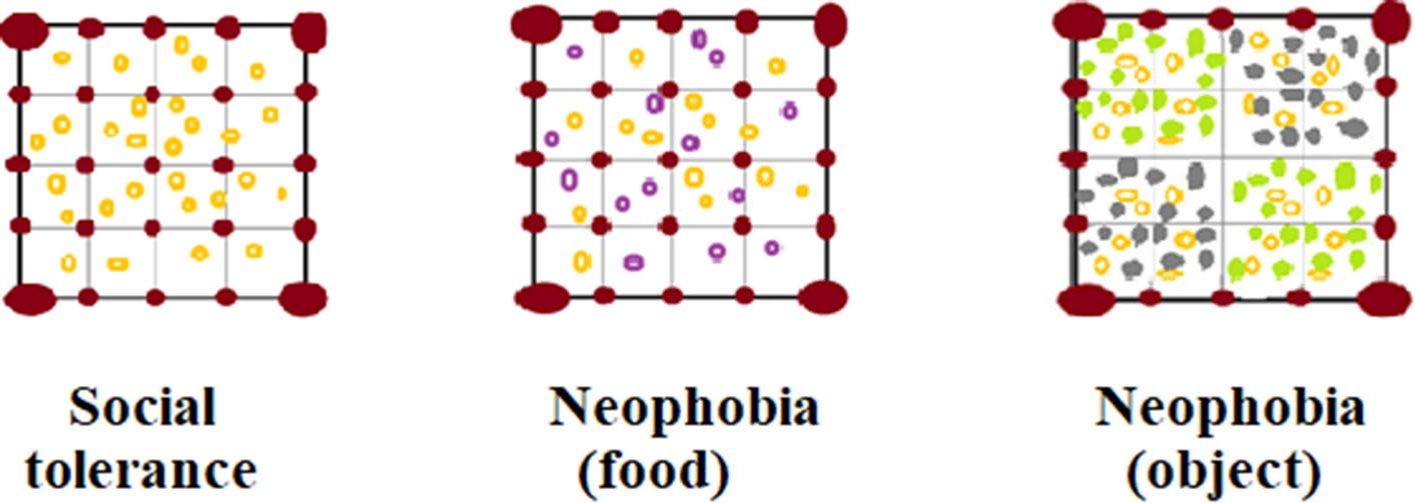
Set-up of the social tolerance task and the neophobia task (object and food conditions). Yellow circles represent familiar food pieces, and purple circles represent novel food pieces (i.e. dyed food). Green marks represent familiar objects (i.e. natural leaves), and grey marks represent novel objects (i.e. dyed leaf-shaped pieces of salt-dough).

During the study period, on designated days each group was administered a social tolerance task, followed by a neophobia task. In both tasks, animals were tested in a testing arena, which consisted of a 4 m × 4 m square area, divided into 4 identical 1 m × 1 m squares marked with stones or branches taken from the local natural environment (Fig. 1). The testing arena was set up in a flat area with little to no vegetation (so that the testing arena was clearly visible), but where the groups naturally foraged on a daily basis.

In the social tolerance task (Fig. 1), subjects were administered 60 sessions per species. Although we aimed to administer 10 sessions a day per group, this was not always possible (i.e., when monkeys left the testing arena to forage in other areas), so the number of testing days varied across groups (JM: 6 days; LM and BM: 7 days each; MM: 9 days). When the group was within view (i.e. more than half of the group members were present), the experimenter threw preferred food items in the testing arena. The session started when the first monkey entered the testing arena. Food consisted of banana slices for all groups, except for JM, who were tested with slices of sweet potato due to dietary restrictions at Koshima. To ensure that this difference had no effect on our results, we re-ran our analyses (see below) after removing JM from the data-set. Results were similar, confirming the significant effect of the same predictors (see Supplementary Materials). Food was distributed evenly throughout the testing arena, and was proportional to the number of adult individuals in each group (i.e., number of adults in the group × 0.4, rounded to the closest even number). However, the number of food pieces retrieved by the macaques did not always coincide with the number of food pieces distributed in the testing arena by the experimenter, either because some pieces were not retrieved (e.g. if they inadvertently got covered) or because some pieces were taken by several individuals (e.g. the pieces were dropped and broken, so that more individuals sampled the original piece). The trial ended when the last piece of food was eaten, if no monkey was in the testing arena for more than 30 s, or if no monkey retrieved food from the testing arena for more than 30 s. At the end of each session, the testing arena was cleaned and new food was distributed throughout, with an interval of at least 5 min between sessions. All group members participated in at least one session of the task.

In the neophobia task, we administered two different conditions (i.e., food and object conditions), to assess reactions to novelty in two different contexts (Fig. 1). The procedure was identical to the social tolerance task, with the following exceptions. In the food condition, half of the food pieces had previously been dyed with a novel food-colour (i.e., red in half of the sessions, and blue in the other half, to maintain novelty high), having no odour and no taste. In the object condition, we first distributed local leaves familiar to the monkeys in two non-adjacent 2 × 2-m squares of the testing arena, and novel coloured pieces of salt-dough in the other two squares. These novel objects had a leaf-shape and size, but clearly differed from a leaf, by having a clay-consistency and being painted in bright colours (i.e. silver in half of the sessions, and another colour in the other half, the second colour differing across species to ensure that the novel objects did not have the same colour of the real leaves, e.g. because of the season). Then, we distributed food as in the social tolerance task. In this way, we could simultaneously present both novel and familiar stimuli to the study subjects, and thus obtain a more controlled measure of neophobia, by measuring the number of familiar versus novel food items retrieved by each individual in each session (see below). We administered 40 sessions per species and condition, starting with 20 food sessions followed by 20 object sessions, and then 20 further food sessions followed by 20 object sessions (using different colours). We used two different conditions, and two different stimuli per condition to create more accurate measures of novelty response^62^, and short sessions to avoid habituation to the novel stimuli^29^.

All sessions were video-recorded. During each session, the experimenter named each individual entering the testing arena, so that it was later possible to code from the videos: (i) which individual entered the testing arena in each session, (ii) how many food pieces were eaten by each individual per session, and (iii) whether the food retrieved was familiar/novel (in the food condition) or collected in the squares with familiar/novel objects (in the object condition).

### Statistical analyses

To avoid the usual top-down approach to the study of dominance styles (i.e. using pre-determined classifications of dominance style for the study species), we directly assessed the dominance styles of the study groups by measuring the steepness of their dominance hierarchy^63^. We used the package steepness (version 0.2-2;^64^) in R^65^ to assess the steepness of the hierarchy in each species, which is considered a central measure of dominance style^4,58^. Steepness was calculated as the absolute value of the slope straight line fitted to the normalized David’s scores, calculated from the proportions of wins in dyadic agonistic interactions^63^. This is considered a more appropriate measure when overall observation time varies between study groups, and actors and recipients interact consistently but infrequently, and is expected to be higher in more despotic species^66^. However, steepness might decrease when the proportion of unknown relationships in a group increases^67^. As the proportion of unknown relationships differed across species (i.e. JM: 51%; LM: 49%; BM: 57%; MM: 62%), we randomly removed dyads with known relationship from the JM, LM and BM dominance matrices, until we reached the same proportion of unknown relationships across all species (i.e. 62%). We then averaged the values obtained over 1000 iterations, and finally assessed the adjusted steepness of the dominance hierarchy for all study species (hereafter, steepness; see^67^).

We then constructed generalized linear models (GLMs) and generalized linear mixed models (GLMMs^68^) with the glmmTMB package (version 1.0.1^69^) in R^65^ to analyse our data. First, we tested whether the proportion of individuals retrieving food was higher in less despotic species (Prediction 1). To test this prediction, we used a binomial GLM to assess whether the relative number of individuals retrieving food in each session of the social tolerance task (versus the number of those retrieving no food, using the ‘cbind’ function) was predicted by the steepness of the species, while controlling for session number (model 1). As we entered one line per session and species, each data point was an independent observation, and no random factors were included.

Second, we tested whether dominance rank and Eigenvector centrality differently affected the probability to retrieve food, depending on the species’ dominance styles (Prediction 2). To test this prediction, we ran a binomial GLMM with the relative number of food pieces retrieved by each subject in each session of the social tolerance task (versus the number of food pieces not retrieved, using the ‘cbind’ function in R^65^) as the dependent variable. In the full model, we included as test predictors the 2-way interactions of steepness with rank and steepness with Eigenvector centrality, along with their main effects. We further included age class, sex and session number as control predictors, and subject identity as a random factor (model 2).

Finally, we tested whether rank and Eigenvector centrality differently affected neophobia (i.e. the probability to retrieve familiar rather than novel food), depending on the species dominance styles (Prediction 3). Accordingly, we used a binomial GLMM to assess whether the relative number of familiar versus novel food items retrieved by each individual in each session of the neophobia task (using the ‘cbind’ function) was affected by the 2-way interactions of steepness with rank and steepness with Eigenvector centrality, along with their main effects. We further included sex, age class, session number and condition (i.e., novel food or novel object) as control predictors, and subject identity as random factor (model 3).

We used likelihood ratio tests^70^ to compare full models containing test, control predictors and random factors, with null models containing only control predictors and random factors using the anova function. If full models were significantly different from null models, we conducted likelihood ratio tests ad used the R function drop1^71^ to obtain the *p* values for each test predictor via single-term deletion. If the 2-way interactions were not significant, we ran the full model again, after removing the interaction and only leaving the main effects. To rule out collinearity, we assessed the VIFs^72^, which were low (maximum VIFs across all models = 1.86). This indicated no collinearity between our test predictors. We detected no convergence issues in any of the models.

## Results

The steepness of the dominance hierarchy varied across species largely in line with what was predicted from literature (steepness: JM: 0.280; LM: 0.290; BM: 0.244; MM: 0.163; adjusted steepness: JM: 0.255; LM: 0.259; BM: 0.234; MM: 0.163). As expected, the least despotic species, MM, had the lowest steepness. However, the difference in steepness for the other three species were less pronounced. Steepness was unexpectedly higher in LM than in JM, but only slightly so.

**Figure 2:**
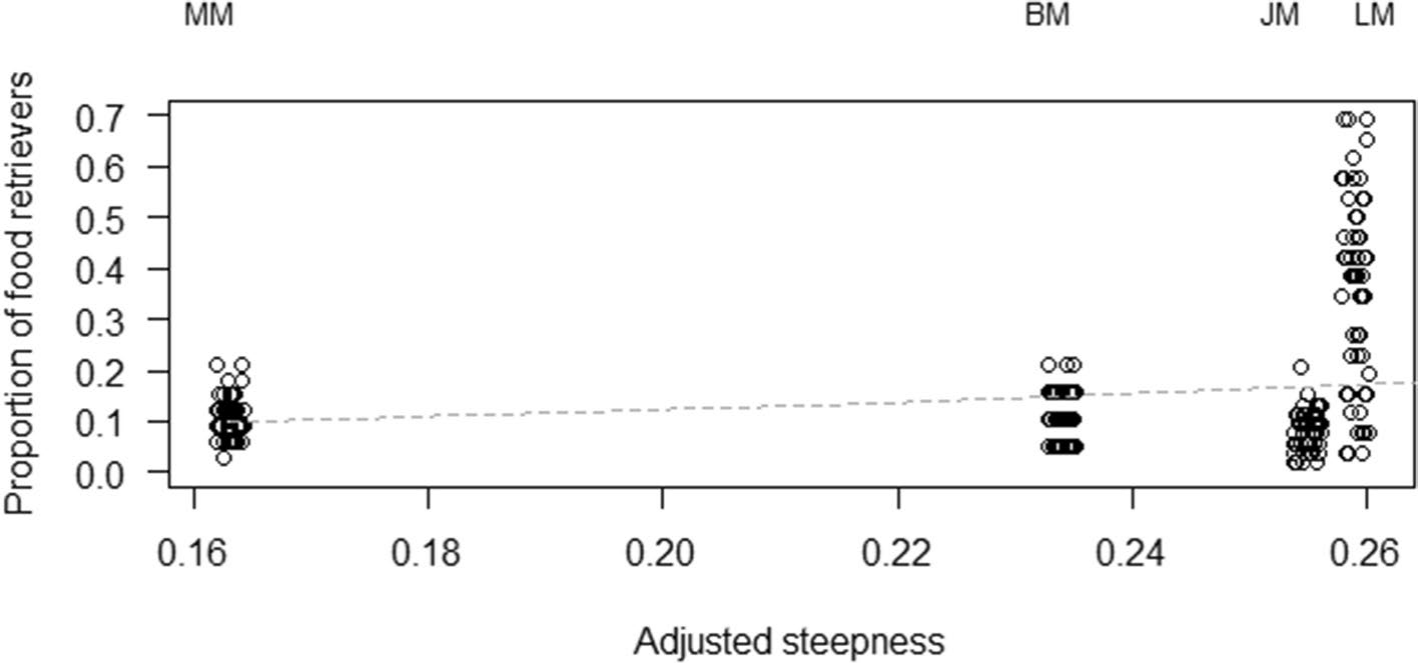
Mean proportion of food retrievers in the social tolerance task, as a function of the steepness of the species (see model 1). Circles represent sessions and are jittered to avoid overlap. The dashed line depicts the model, which has been back-transformed from the log-odds ratio scale and contains standardized controls.

The proportion of individuals retrieving food in each session was highest in LM (0.36 ± 0.19), and lowest in JM (0.08 ± 0.04), with BM and MM showing intermediate levels (0.12 ± 0.05 and 0.11 ± 0.04, respectively). In model 1, we tested whether the proportion of individuals retrieving food was higher in less despotic species (Prediction 1). The full model significantly differed from the null model (GLM: *χ*^2^ = 56.75, df = 1, *p* < 0.001), with a higher steepness predicting a higher proportion of individuals retrieving food (*p* < 0.001; Table 1; Fig. 2).

**Table 1:** Results of models 1 to 3, including estimates, standard errors (SE), z-values (*z*), confidence intervals (CIs), likelihood ratio tests (LRT), degrees of freedom (df), and *P* values for each test and control predictor (in parentheses, the reference category).

Model 1	Estimate	SE	*z*	2.5% CI	97.5% CI	LRT	*df*	*P*
Intercept	− 3.13	0.22	− 14.79	− 3.75	− 2.87	–	–	–
Steepness	6.54	0.91	7.21	4.76	8.32	56.75	1	< 0.001*
*Session*	0.00	0.00	0.66	0.00	0.00	0.43	1	0.510

Model 2	Estimate	SE	*z*	2.5% CI	97.5% CI	LRT	*df*	*P*
Intercept	− 9.29	1.00	− 9.32	− 11.23	− 7.33	–	–	–
Steepness	− 6.56	4.07	− 1.61	− 14.55	1.43	2.57	1	0.109
Rank	4.83	1.06	4.53	2.74	6.91	19.38	1	< 0.001*
Centrality	6.11	0.69	8.81	4.75	7.47	73.16	1	< 0.001*
*Age (Juvenile)*	0.24	0.48	0.49	− 0.70	1.17	0.27	2	0.875
*Age (Subadult)*	0.16	0.49	0.33	− 0.80	1.11			
*Sex (Male)*	0.50	0.35	1.42	− 0.19	1.20	2.00	1	0.158
*Session*	0.00	0.00	0.16	0.00	0.00	0.03	1	0.871

Model 3	Estimate	SE	*z*	2.5% CI	97.5% CI	LRT	*df*	*P*
Intercept	− 1.30	1.20	− 1.09	− 3/65	1.05	–	–	–
Steepness * centrality	− 17.86	8.63	− 2.07	− 34.77	− 0.95	4.20	1	0.040*
Steepness	4.66	5.29	0.88	− 5.72	15.03	–	–	–
Centrality	4.52	2.09	2.16	0.42	8.62	–	–	–
Rank	0.24	0.41	0.58	− 0.57	1.05	0.34	1	0.560
*Condition*	− 0.66	0.06	− 11.03	− 0.78	− 0.54	123.62	1	< 0.001
*Age (Juvenile)*	0.02	0.19	0.10	− 0.35	0.39	1.35	2	0.509
*Age (Subadult)*	0.20	0.18	1.08	− 0.16	0.55			
*Sex (Male)*	0.08	0.14	0.55	− 0.19	0.35	0.30	1	0.582
*Session*	0.00	0.00	− 0.75	0.00	0.00	0.56	1	0.453

Subject identity was included as a random factor in models 2 and 3. The asterisks denote significant *p* values for the test predictors. Control predictors are in italics.

**Figure 3:**
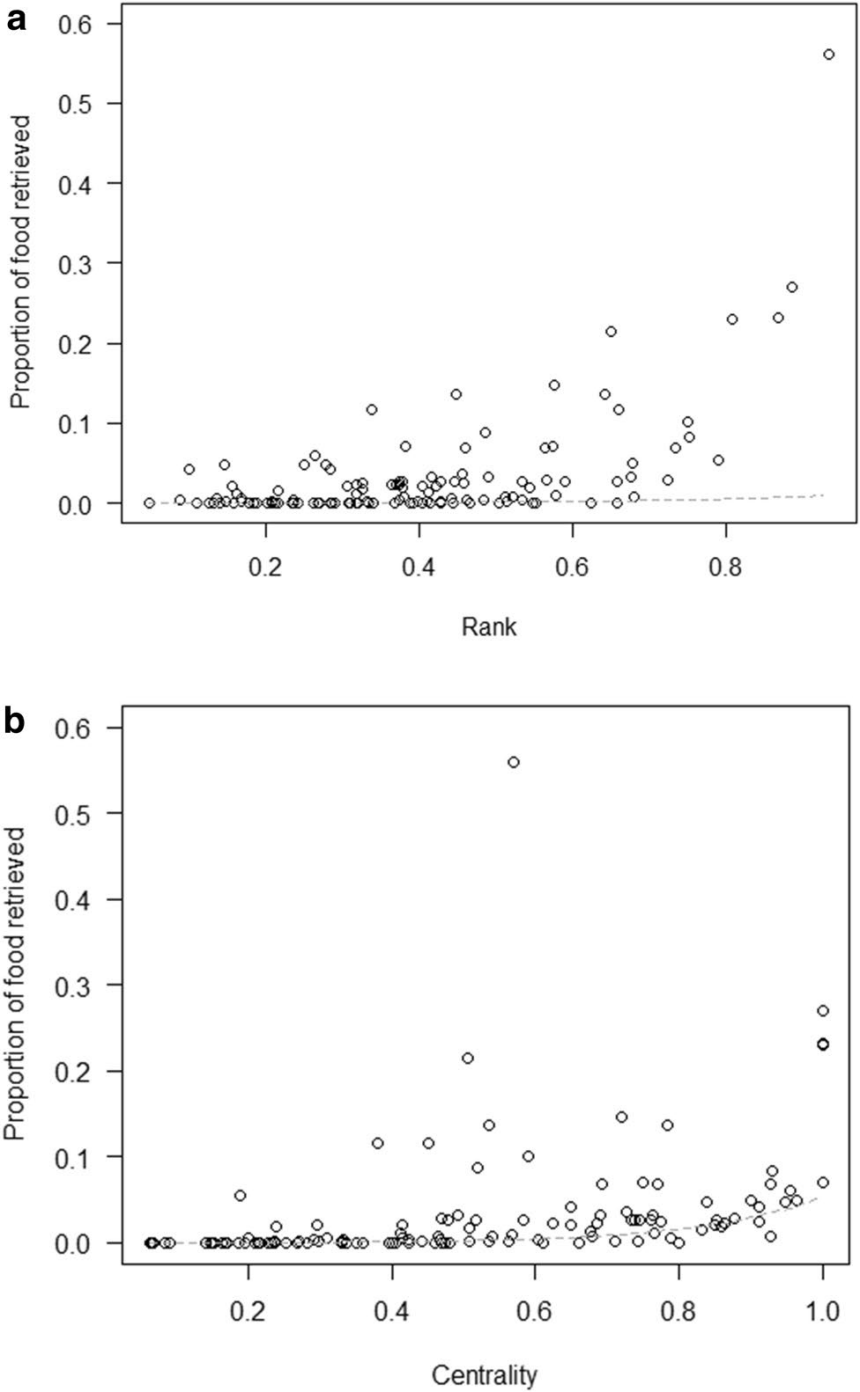
Mean proportion of food retrieved by the study subjects in the social tolerance task, as a function of their (**a**) rank and (**b**) Eigenvector centrality (see model 2). Circles represent the study subjects and are jittered to avoid overlap. The dashed line depicts the model, which has been back-transformed from the log-odds ratio scale and contains standardized controls.

In model 2, we tested whether dominance rank and Eigenvector centrality differently affected the probability to retrieve food, depending on the species dominance styles (Prediction 2). The full-null model comparison was significant (GLMM: *χ*^2^ = 118.46, df = 5, *p* < 0.001), but none of the 2-way interactions were. However, a higher rank and higher Eigenvector centrality both predicted a higher proportion of food retrieved, in all species (both *p* < 0.001; Table 1; Fig. 3a,b).

**Figure 4:**
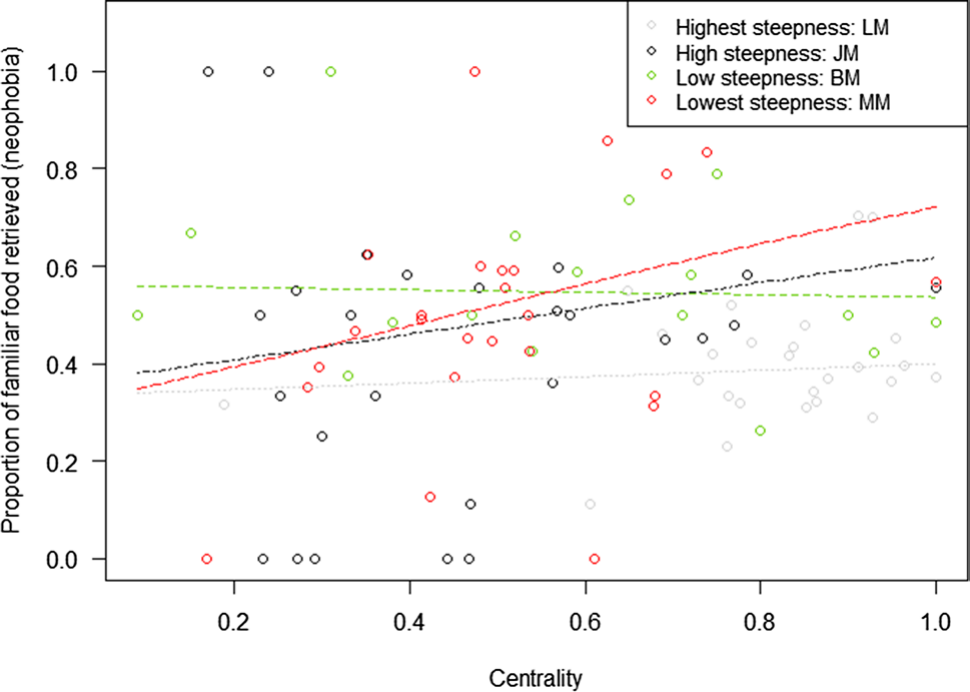
Mean proportion of familiar food retrieved by the study subjects in the social tolerance task (out of the total food retrieved by the subjects, as a measure of neophobia), as a function of their steepness and Eigenvector centrality (model 3). Circles represent the study subjects and are jittered to avoid overlap. Therefore, more neophobic subjects are those depicted in the upper part of the graph. The dashed lines depict the models, which have been back-transformed from the log-odds ratio scale and contain standardized controls.

In model 3, we finally tested whether rank and Eigenvector centrality differently affected neophobia, depending on the species dominance style (Prediction 3). The full-null model comparison was significant (GLMM: *χ*^2^ = 23.16, df = 8, *p* = 0.003). We also found a significant 2-way interaction between steepness and Eigenvector centrality (*p* = 0.031; Table 1): less central individuals were less neophobic (i.e. they were less likely to consume a higher proportion of familiar food), but only in species with lower steepness (Fig. 4).

## Discussion

In this study, we found that different dominance styles^4,5^ only partially predicted the way in which food resources were divided across group members. Firstly, the proportion of individuals accessing food in each session was higher in more despotic species (i.e. with higher dominance steepness), in contrast with our Prediction 1. Secondly, more dominant and more central (i.e. socially integrated) individuals had a higher probability of retrieving food, but this effect was similarly strong in all species, regardless of their dominance steepness (only partially in line with Prediction 2). Finally, less central individuals (who may otherwise have little access to food) were more likely to overcome neophobia than central ones (partially in line with Prediction 3), but only in more despotic species (in contrast with Prediction 3).

Our results suggest that dominance styles have an unexpected effect on how food resources are distributed across group members. In less despotic species, individuals had a lower share of resources (Model 1), and individuals with higher rank and Eigenvector centrality were more likely to retrieve food, similarly to what was found in more despotic species (Model 2). Less despotic species may have less intense aggressive patterns^3,4,8^, but not higher tolerance over food. Alternatively, inter-specific differences in dominance styles may imply a higher intra-specific variation than previously thought, with different aspects of dominance style (e.g. steepness of the dominance hierarchy, occurrence of counter-aggression, tolerance over food) also varying across conspecific populations and not necessarily correlating with each other^63,73,74^. Long-tailed macaques (LM), for instance, are usually considered a moderately despotic species in the literature^4,5^, but in this study their dominance steepness was higher than our study group of Japanese macaques (JM), while their tolerance over food was higher than that of the other three species.

Demographic characteristics of the study groups do not seem to explain these results: Although LM had the highest proportion of juveniles and subadults in the group, which could explain the unexpected high tolerance over food, the results did not change when only analysing the proportion of adults retrieving food (see Supplementary Materials). Alternatively, the specific socio-ecological factors experienced may determine whether despotic tendencies and/or other aspects of dominance style (e.g. tolerance over food) really emerge in a given group. Even the degree of cohabitation with humans and experience in urban habitats may affect the way individuals behave (see e.g.^73^). Although all the study groups were used to humans and partially food-provisioned, the group of LM was living in a more urban area, which might have affected their unexpectedly high tolerance over food (e.g. by providing easier access to food sources, and thus reducing competition over food). Whether this is the case, however, remains at the moment pure speculation. Moreover, one should note that previous studies assessing dominance styles in macaques found a high consistency within species, even if they were tested in very different settings (e.g. captive and wild ones; see^4,75^). Finally, it is interesting to note the larger variation in the proportion of food retrievers in LM, as compared to the other species (Fig. 2). In some sessions, only few LM individuals were present (while the other group members were visible just outside the testing arena). In other sessions, however, almost all the group members were simultaneously inside the testing arena. However, there was no apparent reason for this variation, which was also not linked to the specific identity of the individuals in the testing arena (e.g. higher-ranking individuals could be completely alone in some sessions, or surrounded by many other group members in others).

In our study, the effect of Eigenvector centrality on neophobia also varied across individuals and species, based on their dominance steepness. When re-analysing the data on neophobia by only including the first 6 sessions (to ensure that subjects were not yet habituated to the “novel” stimuli; see Supplementary Materials), the results did not change. In particular, our study showed that less central individuals were less neophobic, but this pattern was only evident in less despotic species. This seems to contrast with our prediction, that the tendency to overcome neophobia is a response of individuals who would otherwise have little access to food to increase food intake. If this was true, neophobia should decrease in less central individuals, as we showed, but mostly in despotic species, as competition over food should be fiercer in these species (see Prediction 3). However, note that, in this study, more despotic species were also the ones with a higher proportion of individuals retrieving food. Therefore, it is possible that, in the presence of novel food, less central individuals more often had to rely on novel food sources to get a share of resources, but only in the groups which showed little tolerance over food (regardless of their dominance steepness). In this view, our Prediction 3 appears confirmed, in that less central individuals more strongly relied on novel food when access to food was more uneven across group members (i.e., surprisingly, in less despotic species). Therefore, although less central individuals might not have a real preference for novelty, specific patterns of tolerance over food would foster them to use alternative retrieval strategies, as they might otherwise have little opportunities to access food.

Our findings can also be explained by the fact that little neophobia may provide different benefits to individuals in different species. In more despotic species, like JM, females inherit their maternal rank, so there may be little incentive for them to be attracted by novelty^76^. However, reduced neophobia in a feeding context may correlate with other traits (e.g. general extraversion, boldness) allowing females in other species to attain other benefits, like higher rank. Therefore, in less despotic species, individuals may more likely overcome neophobia, as this might provide higher benefits.

In the future, these hypotheses should be contrasted to understand whether differences in neophobia are better explained by inter- and intra-specific differences in access to food or personality traits that might favour rank advancement in species with little maternal rank inheritance. Furthermore, future studies should better disentangle the role played by the presence of more dominant individuals on subjects’ performance in neophobia tasks. In this study, we focused on individuals’ ability to overcome neophobia in order to maximize food income. In the future, however, it could be interesting to compare this ability to individual preference for novel food in the absence of contest competition (e.g. by comparing individual preferences for novel food when individuals are tested alone or in group). Moreover, future studies should ideally include a larger variety of novel stimuli. Although in this study we used two different conditions and two different novel stimuli per condition, several species are known to strongly vary in their reactions to different novel stimuli. Thus, using a wider range of stimuli would allow a test of the consistency of our findings^62^. This is especially important if one considers that novelty is after all a matter of degree: no stimulus can be completely novel, as it necessarily shares features with other already familiar stimuli. Finally, differences across study groups might also depend on the different experience of the study groups in urban habitats, as neophobia is known to decrease in urban environments (see^73^).

The results of our study confirm that, beyond dominance rank, other individual characteristics like social integration (measured as Eigenvector centrality in the social network) may have a crucial effect on individuals’ ability to access resources. Although the role of social integration in access to food has rarely been investigated, these results are in line with a recent study on captive Guinea baboons (*Papio papio*^22^) and support a clear link between social integration and fitness in both human^77,78^ and non-human primates^50–52,54,55^.

Overall, our study revealed both inter- and intra-specific differences in individuals’ access to food, which partially reflected the dominance styles of the species examined, and in unexpected ways. In the future, studies will need to directly assess other social aspects of the study groups that might be linked to differences in dominance styles (e.g. agonistic interactions, conflict management patterns, nepotism, availability of social partners, tolerance over food), and the socio-ecological factors that might explain these differences, both across and within species. Further studies should also investigate the role of Eigenvector centrality in access to food, to better understand the multiple effects that sociality may have on fitness, and better disentangle possible differences between male and female behaviour, as differences in dominance style should mainly affect females^4,5^.

## Supplementary information

Supplementary Information 1.

Supplementary Information 2.
