# Supplementary material for: Dominance style only partially predicts differences in neophobia and social tolerance over food in four macaque species
Source: Sci Rep. 2020 Dec 16;10:22069. doi: 10.1038/s41598-020-79246-6 (PMC7744554; doi:10.1038/s41598-020-79246-6)
Supplement: Supplementary file 2 — Supplementary Information 2. [file 41598_2020_79246_MOESM2_ESM.docx]

TITLE: Dominance style only partially predicts differences in neophobia and social tolerance over food in four macaque species

AUTHORS: Federica Amici ^1,2^, Anja Widdig ^1,2^, Andrew JJ MacIntosh ^3^, Victor Beltrán Francés ^4^, Alba Castellano-Navarro ^5^, Alvaro Lopez Caicoya ^6^, Karimullah Karimullah ^1,7^, Risma Illa Maulany ^8^, Ngakan Putu Oka ^8^, Andi Siady Hamzah ^8^, Bonaventura Majolo ^9^

^1^ Behavioral Ecology Research Group, Institute of Biology, Faculty of Life Science, University of Leipzig, Germany

^2^ Research Group Primate Behavioural Ecology, Department of Human Behavior, Ecology and Culture, Max-Planck Institute for Evolutionary Anthropology, Leipzig, Germany

^3^ Primate Research Institute, Kyoto University, Aichi, Japan

^4^ Fundació Universitat de Girona, Innovació i Formació, Girona, Spain

^5^ Ethology and Animal Welfare Section, Universidad Cardenal Herrera-CEU, CEU Universities, Valencia, Spain

^6^ Department of Clinical Psychology and Psychobiology, Faculty of Psychology, University of Barcelona, Spain

^7^ School of Biological Sciences, Universiti Sains Malaysia, Pulau Pinang, Malaysia

^8^ Forestry Department, Hasanuddin University, Makassar, Sulawesi, Indonesia

^9^ School of Psychology, University of Lincoln, United Kingdom

CORRESPONDING AUTHOR: Federica Amici; amici@eva.mpg.de

**SUPPLEMENTARY MATERIAL**

**METHODS**

*Subjects.* The study groups differed in their group composition in term of sex, age class, rank and integration in the social network (Table S1), having a different proportion of adults and sexes in each group (Table S2). Japanese macaques (JM) were studied on Koshima Islet, Miyazaki prefecture, Japan (31°27′N, 131°22′E). The island is inhabited by two groups, and by solitary males. We studied the Main group, which was well habituated and lived in an evergreen broadleaved forest, with access to the beach. More details on the socio-ecology of the group can be found in the abundant literature that has been published on this group (e.g. [1-3]). The long-tailed macaques (LM) were studied in the suburbs of Kuala Lumpur, in Malaysia (5”18”48.98 N, 100”24”38,84 E). The study group lived in an area with mangrove forests, rice crops and villages. To our knowledge, no study had been ever conducted on this group. The Barbary macaques (BM) were studied in Gibraltar (36°08'37.5"N 5°20'36.5"W). The group lived in an area characterized by steep cliffs and sparse vegetation, in a military zone where tourists and inhabitants have no access. Finally, the moor macaques (MM) were studied in the Karaenta forest area, Bantimurung-Bulusaraung National Park, South Sulawesi, Indonesia (5°02’59.4” S, 119°44’13.2” E). The group was well habituated and lived in an area characterized by primary forest, in the karst ecosystem of the area. The group we observed was well habituated, having been studied for more than 30 years (e.g. [4-6]).

In all study groups, monkeys foraged and fed mainly on natural food. However, all study groups were also partially provisioned with small quantities of fruit and vegetables cut in small pieces by humans other than the experimenters, which could not be easily monopolized by one single monkey. In particular, JM on Koshima island were fed twice a week by local researchers; BM in Gibraltar were provided with food on a daily basis by a local NGO; and LM and MM were provided with food by occasional local tourists in Malaysia and Sulawesi. As individuals were free to participate in the testing sessions, the number of individuals in each study group does not necessarily correspond to the number of individuals participating in the tasks.

*Materials and procedures.* Behavioural observations were conducted on JM from November 2016 until June 2017; on LM from October 2018 until March 2019; on BM from October 2017 until March 2018; and on MM from September 2018 until March 2019.

*Statistical analyses.* Together with the models described in the main text, we also ran three further models. Model A was similar to model 1 in the main text, and was run to assess whether the proportion of individuals retrieving food varied across species depending on the steepness of their dominance hierarchy, when only including adults in the model. We decided to run this model, because LM showed an unexpectedly high proportion of individuals retrieving food (see model 1 in the main text). Given that LM were also the study group with the highest proportion of juveniles and subadults (see Table S2), this further model on only adults allowed us to rule out that the unexpected proportion of food retrievers in LM (model A) was due to the presence of more juveniles and subadults. To run this new model, we first excluded juveniles and subadults from the data-set, and then separately assessed the relative number of females and males retrieving food in each session of the social tolerance task, versus the number of females and males retrieving no food. We then used a binomial GLMM to assess whether the relative number of adults retrieving food versus the number of those retrieving no food (using cbind) was affected by the interaction of steepness and sex (also including them as main effects). As we entered two lines per session (i.e. one for females, one for males), session number was included here as a random factor.

Model B was also similar to model 1 in the main text, but was run after removing data on JM. JM were the only species having been tested with a different food than the other three species (i.e. sweet potatoes instead of bananas). So, by removing them from the analyses we could check whether the results would remain the same. This model included the same variables as model 1: a binomial GLM, with the relative number of individuals retrieving food in each session (versus the number of those retrieving no food, using the ‘cbind’ function) as dependent variable, steepness as test predictor, and session number as control.

Finally, model C was similar to model 3 in the main text, but for each study subject we only included the first 6 sessions of the neophobia task, in order to ensure that subjects had not yet habituated to the novel stimuli. The model was otherwise identical to model 3: a binomial GLMM, with the relative number of familiar versus novel food items retrieved by each individual in each session of the neophobia task (using the ‘cbind’ function) as dependent variable, the 2-way interactions of steepness with rank and steepness with Eigenvector centrality as test predictors (along with their main effects), sex, age class, session number and condition as controls, and subject identity as random factor.

**RESULTS**

Model A revealed a significant difference between the full and null models (GLMM: *χ*^2^ = 209.51, df = 3, *p* < 0.001), with the relative number of adults retrieving food in each session being predicted by the 2-way interaction of steepness and sex (*p* < 0.001; see Table S3). In particular, the relative number of adults retrieving food was higher when the steepness was higher, and this effect was stronger in females than in males. Therefore, these results confirm those reported in the Main text for model 1.

Similarly, the full and null models differed for model B (GLMM: *χ*^2^ = 261.37, df = 1, *p* < 0.001), with a higher steepness predicting a higher proportion of individuals retrieving food (*p* < 0.001; see Table S4). Therefore, these results confirm those reported in the Main text for model 1.

Finally, model C also revealed a significant difference between the full and the null models (GLMM: *χ*^2^  = 14.86, df = 5, *p* = 0.011). In particular, the 2-way interaction of steepness and Eigenvector centrality was significant (see Table S5). As in the model 3 in the main text, therefore, less central individuals were less neophobic (i.e. they were less likely to consume a higher proportion of familiar food), but only in species with lower steepness.

**REFERENCES**

Kawai M. 1965 Newly acquired pre-cultural behavior of the natural troop of Japanese monkeys on Koshima Islet. *Primates* **6**, 1-30.

Nakagawa N, Nakamichi M, Sugiura H (eds). 2010 *The Japanese macaques.* Springer Science & Business Media, pp. 6-10.

1. Watanabe K. 1994 Precultural behavior of Japanese macaques: longitudinal studies of the Koshima troops. In Gardner RA, Gardner B, Chiarelli B, Plooij FX (eds). *The ethological roots of culture.* Kluwer, pp 81-94.
2. Matsumura S. 1993 Female reproductive cycles and the sexual behavior of moor macaques (*Macaca maurus*) in their natural habitat, South Sulawesi, Indonesia. *Primates* **34**, 99-103.
3. Matsumura S. 1996 Postconflict affiliative contacts between former opponents among wild moor macaques (*Macaca maurus*). *Am. J. Primatol.* **38**, 211-219.
4. Okamoto, K., & Matsumura, S. (2001). Group fission in moor macaques (*Macaca maurus*). *Int. J. Primatol.* **22**, 481-493.

**Table S1.** List of the subjects tested in this study, with their sex, age, rank and centrality.

| **Species** | **Subject** | **Sex** | **Age class** | **Rank** | **Centrality** |
| --- | --- | --- | --- | --- | --- |
| Barbary macaques  (BM) | Batmana | female | adult | 0.566 | 0.47 |
|  | Chicho | male | juvenile | 0.678 | 0.90 |
|  | Colega | male | adult | 0.307 | 0.65 |
|  | Grunongibraltar | male | adult | 0.660 | 0.38 |
|  | Jefa | female | adult | 0.576 | 0.72 |
|  | Legolashijo | male | adult | 0.442 | 0.20 |
|  | Legolaspadre | male | adult | 0.752 | 0.93 |
|  | Mamabebeenano | female | adult | 0.048 | 0.66 |
|  | Mamanoel | female | adult | 0.681 | 0.54 |
|  | Mancha | female | adult | 0.624 | 0.15 |
|  | Mephisto | male | adult | 0.750 | 0.59 |
|  | Nerd | female | adult | 0.341 | 0.09 |
|  | Noruega | female | adult | 0.505 | 0.80 |
|  | Orejacortada | female | adult | 0.382 | 0.75 |
|  | Paul | male | adult | 0.887 | 1.00 |
|  | Pedro | male | juvenile | 0.399 | 0.71 |
|  | Pendiente | male | adult | 0.136 | 0.31 |
|  | Tetas | female | adult | 0.141 | 0.33 |
|  | Uniteta | female | adult | 0.486 | 0.52 |
| Long-tailed macaques  (LM) | Alfaalfa | male | adult | 0.79 | 0.19 |
|  | Alfamal | male | adult | 0.808 | 1.00 |
|  | Amar | male | subadult | 0.522 | 0.93 |
|  | Asa | female | adult | 0.49 | 0.76 |
|  | Bakar | male | subadult | 0.365 | 0.86 |
|  | Beta | male | adult | 0.564 | 0.93 |
|  | Emelie | female | juvenile | 0.147 | 0.84 |
|  | Ethzo | female | adult | 0.535 | 0.76 |
|  | Fahro | male | juvenile | 0.101 | 0.65 |
|  | Gamma | male | adult | 0.458 | 0.91 |
|  | Gulpana | female | adult | 0.447 | 0.75 |
|  | Ida | female | adult | 0.278 | 0.95 |
|  | Ina | female | subadult | 0.456 | 0.73 |
|  | Ju | female | juvenile | 0.374 | 0.78 |
|  | Juri | male | juvenile | 0.216 | 0.83 |
|  | Mizo | female | adult | 0.37 | 0.69 |
|  | Momo | female | adult | 0.59 | 0.85 |
|  | Moon | female | juvenile | 0.319 | 0.77 |
|  | Nivola | female | juvenile | 0.089 | 0.61 |
|  | Shero | male | juvenile | 0.264 | 0.95 |
|  | Su | female | juvenile | 0.379 | 0.86 |
|  | Wahid | male | adult | 0.725 | 0.88 |
|  | Wati | female | subadult | 0.251 | 0.96 |
|  | Zahir | male | juvenile | 0.156 | 0.85 |
|  | Zaid | male | subadult | 0.284 | 0.91 |
|  | Zara | female | juvenile | 0.375 | 0.79 |
| Japanese macaques  (JM) | Beni | female | adult | 0.234 | 0.15 |
|  | Betei | female | subadult | 0.159 | 0.15 |
|  | Binega | female | adult | 0.111 | 0.24 |
|  | Bon | male | subadult | 0.262 | 0.17 |
|  | Botan | female | juvenile | 0.217 | 0.22 |
|  | Hado | female | adult | 0.286 | 0.36 |
|  | Haku | male | juvenile | 0.321 | 0.35 |
|  | Hiba | female | subadult | 0.263 | 0.25 |
|  | Kanna | female | adult | 0.27 | 0.17 |
|  | Kei | male | adult | 0.936 | 0.57 |
|  | Keta | male | adult | 0.658 | 0.07 |
|  | Kibana | female | adult | 0.463 | 0.47 |
|  | Kinoko | female | adult | 0.429 | 0.46 |
|  | Kizu | female | adult | 0.484 | 0.29 |
|  | Komatsu | female | juvenile | 0.287 | 0.47 |
|  | Kote | male | subadult | 0.392 | 0.40 |
|  | Mekki | male | juvenile | 0.203 | 0.17 |
|  | Mikan | female | adult | 0.335 | 0.27 |
|  | Minku | male | adult | 0.552 | 0.17 |
|  | Mizu | female | adult | 0.335 | 0.15 |
|  | Mochi | female | juvenile | 0.209 | 0.22 |
|  | Muku | female | adult | 0.235 | 0.23 |
|  | Mushi | male | adult | 0.445 | 0.09 |
|  | Neji | male | adult |  | 0.00 |
|  | Nire | female | adult |  | 0.01 |
|  | Noko | male | juvenile | 0.319 | 0.21 |
|  | Noru | male | adult |  | 0.07 |
|  | Okapi | male | adult | 0.466 | 0.24 |
|  | Okura | female | adult | 0.544 | 0.24 |
|  | Omoto | female | adult | 0.549 | 0.27 |
|  | Pan | female | juvenile | 0.242 | 0.24 |
|  | Pichi | female | subadult | 0.185 | 0.14 |
|  | Serori | female | adult | 0.291 | 0.23 |
|  | Shida | male | juvenile | 0.375 | 0.73 |
|  | Shide | female | adult | 0.643 | 0.78 |
|  | Shiira | male | subadult | 0.416 | 0.69 |
|  | Shika | male | adult | 0.659 | 0.48 |
|  | Sisho | female | adult | 0.578 | 0.57 |
|  | Suma | male | adult |  | 0.01 |
|  | Tabu | female | subadult | 0.461 | 0.77 |
|  | Toga | female | adult | 0.213 | 0.27 |
|  | Tsutsuji | female | adult | 0.327 | 0.58 |
|  | Tsuwa | female | adult | 0.411 | 0.44 |
|  | Uso | male | adult |  | 0.07 |
|  | Usu | female | adult | 0.284 | 0.20 |
|  | Yamu | female | adult | 0.514 | 0.56 |
|  | Yashi | female | adult | 0.574 | 1.00 |
|  | Yomogi | male | subadult | 0.332 | 0.30 |
|  | Yone | female | adult | 0.368 | 0.40 |
|  | Yotsuba | female | juvenile | 0.237 | 0.33 |
|  | Yumin | male | juvenile | 0.189 | 0.15 |
|  | Yuna | female | adult | 0.31 | 0.19 |
|  | Yuu | male | adult | 0.415 | 0.07 |
| Moor macaques  (MM) | Abuabu | male | adult | 0.677 | 0.49 |
|  | Adinda | female | adult | 0.179 | 0.06 |
|  | Afwan | male | juvenile | 0.512 | 0.47 |
|  | Ale | male | adult | 0.308 | 0.34 |
|  | Alif | male | subadult | 0.428 | 0.52 |
|  | Bb | female | adult | 0.268 | 0.47 |
|  | Betty | female | adult | 0.132 | 0.41 |
|  | Caca | female | adult | 0.326 | 0.51 |
|  | Ciro | female | juvenile | 0.208 | 0.33 |
|  | Cri | female | adult | 0.127 | 0.48 |
|  | Eli | female | adult | 0.405 | 0.07 |
|  | Finny | female | adult | 0.203 | 0.28 |
|  | Hantu | male | adult | 0.535 | 0.42 |
|  | Hendra | male | adult | 0.413 | 0.68 |
|  | Jaya | male | adult | 0.87 | 1.00 |
|  | Kopi | male | adult | 0.449 | 0.54 |
|  | Lani | female | adult | 0.429 | 0.74 |
|  | Lavena | female | adult | 0.163 | 0.41 |
|  | Lem | male | adult | 0.334 | 0.35 |
|  | Lucia | female | subadult | 0.169 | 0.54 |
|  | Moka | female | adult | 0.38 | 0.68 |
|  | Momoa | male | adult | 0.405 | 0.30 |
|  | Nopi | female | adult | 0.29 | 0.61 |
|  | Putri | female | adult | 0.149 | 0.51 |
|  | Rissa | female | subadult | 0.319 | 0.63 |
|  | Rokko | male | adult | 0.236 | 0.17 |
|  | Sahril | female | subadult | 0.168 | 0.41 |
|  | Singa | male | juvenile | 0.339 | 0.45 |
|  | Sugi | female | adult | 0.389 | 0.42 |
|  | Tara | male | subadult | 0.422 | 0.41 |
|  | Titi | female | adult | 0.378 | 0.74 |
|  | Topi | male | adult | 0.651 | 0.51 |
|  | Waps | male | adult | 0.735 | 0.69 |

Sex and age were estimated based on visual and behavioural cues in all groups, except for Japanese macaques (for which demographic and life-history data are regularly collected). Infants (i.e. younger than 1 years of age) are not included in the list. We then classified as juveniles all males between 1 and 3 years of age, and all females between 1 and 2; as subadults all males between 3 and 4 years of age, and all females between 2 and 3; and as adults all males of age 5 or older, and all females of age 4 or older. One corresponds to high rank, and 0 to low rank. Similarly, 1 corresponds to high and 0 to low centrality.

**Table S2.** For each species, number of juveniles and subadults, adult males and adult females, and their proportion relative to the total number of individuals in the group.

| **Species** | **Juveniles & subadults** | | **Adult females** | | **Adult males** | |
| --- | --- | --- | --- | --- | --- | --- |
|  | N | Proportion | N | Proportion | N | Proportion |
| Japanese macaques (JM) | 18 | 0.34 | 24 | 0.45 | 11 | 0.21 |
| Long-tailed macaques (LM) | 15 | 0.58 | 6 | 0.23 | 5 | 0.19 |
| Barbary macaques (BM) | 2 | 0.11 | 10 | 0.53 | 7 | 0.37 |
| Moor macaques (MM) | 8 | 0.24 | 14 | 0.42 | 11 | 0.33 |

**TABLE S3.** Results of model A (i.e. only including adults), including estimates, standard errors (SE), z-values (*z*), confidence intervals (CIs), likelihood ratio tests (LRT), degrees of freedom (df), and *P* values for the 2-way interaction as test predictor.

| **Model A – only adults** | **Estimate** | **SE** | ***z*** | **2.5% CI** | **97.5% CI** | **LRT** | **Df** | ***P*** |
| --- | --- | --- | --- | --- | --- | --- | --- | --- |
| Intercept | -6.64 | 0.61 | -10.89 | -7.83 | -5.44 | - | - | - |
| Steepness | 18.11 | 2.48 | 7.31 | 13.25 | 22.96 | - | - | - |
| Sex | 5.04 | 0.68 | 7.39 | 3.70 | 6.38 | - | - | - |
| Sex (Male)*Steepness | -17.20 | 2.82 | -6.09 | -22.73 | -11.67 | 44.36 | 1 | <0.001 |

Session number was included as random factor. In parentheses, the reference category is included.

**TABLE S4.** Results of model B (i.e. after excluding Japanese macaques, JM), including estimates, standard errors (SE), z-values (*z*), confidence intervals (CIs), likelihood ratio tests (LRT), degrees of freedom (df), and *P* values for the test predictor and control predictor (the last one in italics).

| **Model B – no JM** | **Estimate** | **SE** | ***z*** | **2.5% CI** | **97.5% CI** | **LRT** | **Df** | ***P*** |
| --- | --- | --- | --- | --- | --- | --- | --- | --- |
| Intercept | -4.67 | 0.24 | -19.62 | -5.14 | -4.21 | - | - | - |
| Steepness | 14.91 | 0.99 | 15.10 | 12.97 | 16.84 | 261.37 | 1 | <0.001 |
| *Session* | 0.00 | 0.00 | -0.55 | -0.01 | 0.00 | 0.31 | 1 | 0.580 |

**TABLE S5.** Results of model C (i.e. only including the first 6 sessions), including estimates, standard errors (SE), z-values (*z*), confidence intervals (CIs), likelihood ratio tests (LRT), degrees of freedom (df), and *P* values for each test and control predictor (in parentheses, the reference category), with control predictors being in italics.

| **Model C – first 6 sessions** | **Estimate** | **SE** | ***z*** | **2.5% CI** | **97.5% CI** | **LRT** | **Df** | ***P*** |
| --- | --- | --- | --- | --- | --- | --- | --- | --- |
| Intercept | -0.57 | 1.11 | -0.51 | -2.74 | 1.60 | - | - | - |
| Steepness * Centrality | -20.58 | 9.40 | -2.19 | -39.01 | -2.15 | 4.79 | 1 | 0.029 |
| Steepness | 0.95 | 4.99 | 0.19 | -8.83 | 10.73 | - | - | - |
| Centrality | 5.02 | 2.30 | 2.18 | 0.50 | 9.53 | - | - | - |
| Steepness * Rank | 13.27 | 9.20 | 1.44 | -4.76 | 31.30 | 2.09 | 1 | 0.149 |
| Rank | -2.68 | 2.25 | -1.19 | -7.10 | 1.73 | - | - | - |
| *Condition* | -0.44 | 0.09 | -5.04 | -0.61 | -0.27 | 25.47 | 1 | <0.001 |
| *Age (Juvenile)* | 0.01 | 0.19 | 0.07 | -0.35 | 0.38 | 2.60 | 2 | 0.272 |
| *Age (Subadult)* | 0.26 | 0.18 | 1.49 | -0.08 | 0.61 |  |  |  |
| *Sex (Male)* | 0.15 | 0.14 | 1.07 | -0.12 | 0.42 | 1.16 | 1 | 0.282 |
| *Session* | -0.01 | 0.03 | -0.34 | -0.06 | 0.04 | 0.116 | 1 | 0.733 |

Subject identity was included as random factor. In parentheses, the reference category is included.
